# Radiographic viewing conditions at Johannesburg Hospital

**DOI:** 10.2349/biij.4.2.e17

**Published:** 2008-04-01

**Authors:** T Nyathi, AN Mwale, P Segone, SH Mhlanga, ML Pule

**Affiliations:** 1 School of Physics, University of the Witwatersrand, Johannesburg, South Africa>; 2 Division of Medical Physics, Johannesburg Hospital, Johannesburg, South Africa

**Keywords:** Luminance, ambient lighting, quality assurance

## Abstract

**Purpose:**

To measure the luminance level of X-ray viewing boxes and ambient lighting levels in reporting rooms as a quality assurance procedure, and to compare the results with those recommended by the Directorate of Radiation Control, South Africa (DRC), European Commission (EC) and Nordic Radiation Protection Co-operation (NORDIC).

**Materials and Methods:**

All the viewing boxes housed at the Divisions of Radiation Oncology and Radiology of Johannesburg Hospital had their luminance levels measured using a calibrated photometer. In addition the room’s ambient light was measured using a photometer.

**Results:**

The mean average luminance was 1026.75 ± 548.65 cd m^-2^ and 3284.38 ± 327.91 cd m^-2^ at the Division of Radiology and Division of Radiation Oncology respectively. The Division of Radiation Oncology had an average viewing box uniformity of 7.14% compared to 27.32% at the Division of Radiology. The average ambient lighting was found to be 66.30 lux and 66.43 lux at the Division of Radiation Oncology and Division of Radiology respectively.

**Conclusion:**

The radiograph viewing conditions in Johannesburg variably comply with guidelines. This study underscores the need to implement quality control and quality assurance standards in radiographic image viewing.

## INTRODUCTION

When light intensity is low, the eye transfers from cone vision to rod vision. This is because rod vision is more sensitive. At low light intensities, the eye loses its resolving power or visual acuity [1]. To achieve optimal visual acuity, it is recommended that the retinal cones receive an incident luminance of 100 cd m^-2^ (candela per square metre) [2].

Given this background it becomes clear that an accurate interpretation of a radiograph is a function of the viewing conditions and it will generally decrease as the viewing conditions deteriorate [2, 3]. The importance of viewing conditions has been overlooked in the process of optimising the diagnostic radiology process, yet the whole radiographic process can only be as strong as its weakest link. Many researchers in radiology have concentrated on dose and image quality optimisation, while ignoring the viewing conditions. A literature survey has shown that five factors contribute to poor film reader performance, namely:

- Suboptimal illumination level.
- Excessive pupil dilation.
- Light scatter within the film.
- View box glare.
- Improper ambient light level [3].

The objective of this study was to measure the luminance level of X-ray viewing boxes and ambient lighting levels in reporting rooms as a quality assurance procedure.

## MATERIALS AND METHODS

This investigation included all the conventional viewing boxes and radiograph viewing areas/reporting rooms in the Divisions of Radiology and Radiation Oncology at Johannesburg Hospital. The viewing box luminance (brightness), viewing box luminance uniformity, and the viewing area ambient lighting were measured using a calibrated Nuclear Associates Precision Photometer, Model 07-621, manufactured by Fluke Biomedical Radiation Management Services. The photometer was calibrated at the Nuclear Associates factory in the United States of America. Measurements were taken at mid-morning, hours after the viewing boxes had been switched on, with the assumption that the viewing box light output would have stabilised at the time of measurement.

To assess the viewing conditions and the ambient lighting, recommendations or guidelines from the DRC, NORDIC (Denmark, Finland, Iceland, Norway and Sweden) and the EC were used as standards [4-6]. These guidelines for the parameters viewing box luminance, uniformity of viewing box and ambient lighting are shown in Table 1.

**Table 1:** Tabulation of published guidelines.

Organisation	Luminance of viewing box (cd m-2)	Uniformity of viewing box (cd m-2)	Ambient lighting (lux)
DIRECTORATE: RADIATION CONTROL	≥ 1500	≤ 20	≤ 100
NORDIC	1500 - 3000	≤ 15	≤ 100
EUROPEAN COMMISSION	≥ 1700	≤ 30	≤ 50

For the purposes of measuring luminance, the view box was divided into four quadrants so that five measurements could be taken, namely at the centre of the view box and also at the centre of each quadrant. These measurements were taken with the photometer positioned flush on the view box. Viewing box luminance uniformity was determined using the relationship given below [7]:

(1) $$Uniformity(\%)=\frac{C_{\max}-C_{\min}}{C_{\max}+C_{\min}}\times 100$$

where C_max_ is the maximum luminance measured and C_min_ is the minimum luminance value measured on the viewing box.

Ambient lighting was measured from a distance of 30 cm away from a switched-off viewing box [7, 8]. It should be noted that the European Commission suggests that the ambient lighting be measured at a distance of 1 m. The measurement distance of 30 cm from the viewing box was adopted for this study since it approximates the distance between the viewer and the viewing box in a typical clinical setting. The unit for ambient lighting is the lux.

## RESULTS

A total of 47 viewing boxes were analysed in this study. From this total, 24 viewing boxes were located in various areas at the Division of Radiology and the remaining 23 were located at the Division of Radiation Oncology. Results of the investigation are tabulated in Table 2.

**Table 2:** Tabulation of the experimental results. The standard deviation in the measured quantity is shown in brackets.

Division	Mean Average Luminance (cd m-2)	Mean Central Luminance (cd m-2)	Average Uniformity (%)	Average Ambient Lighting (lux)
Radiology	1026.75 (548.65)	1285.00 (666.30)	27.32 (12.51)	66.43 (25.21)
Radiation Oncology	3284.38 (327.91)	3304.76 (407.02)	7.14 (4.33)	66.30 (31.91)

**Figure 1:**
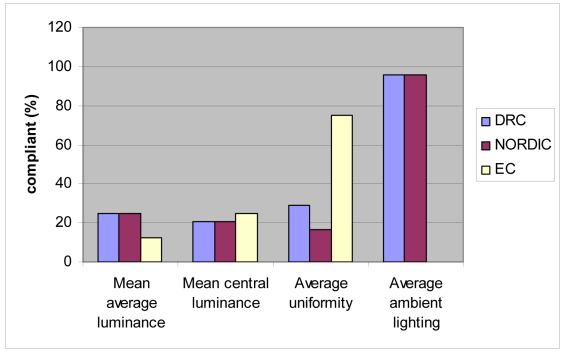
Bar chart shows the percentage of viewing boxes at the Division of Radiology compliant to different guidelines.

**Figure 2:**
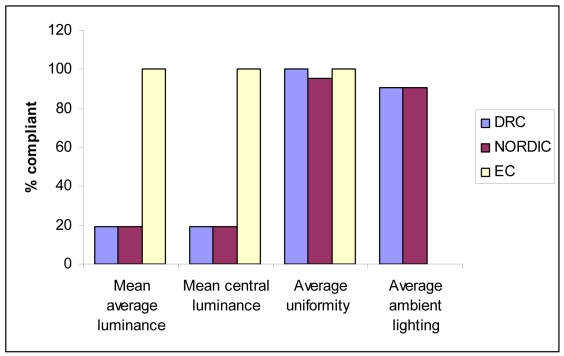
Bar chart showing percentage of viewing boxes at the Division of Radiotherapy complying with different guidelines.

The spread in the magnitude of mean average luminance, mean central luminance, average uniformity and ambient lighting was quantified by the standard deviation. The data in Table 2 was compared with the published guidelines from the DRC, NORDIC and the EC. Figures 1 and 2 show the percentage of viewing boxes which were in compliance with the guidelines as set out by different organisations at the Divisions of Radiology and Radiation Oncology respectively.

## DISCUSSION

If the whole radiographic process chain is to be fully optimised, it becomes imperative for viewing box luminance and ambient lighting to be optimal. Maintaining optimal radiograph viewing conditions is simple and cheap to achieve. It is thus recommended that optimum radiograph viewing conditions be in place in order for the radiologist to get the most diagnostic information from each radiograph. The three organisations’ recommendations on the measured quantities in this study are not in total agreement; as such it would be a good idea for the radiology community to harmonise these guidelines worldwide or alternatively adopt one set of guidelines from one organisation.

Radiologists and radiation oncologists at Johannesburg Hospital continue to use these viewing boxes for reporting despite the fact that these do not comply with the guidelines. Admittedly the detrimental effect of non-optimal viewing conditions is not as pronounced in general radiography as it is in mammography. However, there are studies which have proved that non-optimal viewing conditions affect the radiologist’s ability to detect low contrast lesions [9, 10]. In addition a number of studies have shown that dental radiography is affected by non-optimal viewing conditions [11, 12]. The effect of the continual use of non-compliant viewing boxes can be determined by conducting observer performance studies or psychophysical experiments.

After 2000 hours of use, the luminance of fluorescent tubes decreases by approximately 10% [3]. The American College of Radiology (ACR) recommends replacement of fluorescent tubes after every 18 to 24 months. This could explain the high level of viewing box luminance uniformity at the Division of Radiation Oncology as the division has just been recently opened.

The European Commission recommends that the ambient lighting be measured at a distance of 1 m, thus compliance with the European guidelines was not analysed. In this present study, ambient lighting was measured a distance of 30 cm, this being premised on the fact that a distance of 30 cm approximates the distance between the viewer and the viewing box in a clinical setting, as one would rarely view a radiography at a distance of 1 m. Since the viewing box should be switched off during measurement, it is expected that ambient lighting should not vary with measurement distance.

The average luminance is a better indicator of the viewing box luminance than the central luminance. This is confirmed by the greater standard deviation in the mean central luminance than the standard for the mean average luminance as shown in Table 2.

Screen-film technology still has widespread use in developing countries and Johannesburg Hospital is no exception. As such it is imperative to have quality control measures on viewing boxes. Despite the widespread use of screen-film technology in developing countries, there is a gradual shift to digital X-ray systems, which could be the ultimate solution to non-optimal viewing box luminance having a detrimental effect on radiograph reporting. The use of digital systems paves the way for other viewing options like the use of video monitors, printing the image on paper and the use of picture archiving and communication systems (PACS), which have the advantage of eliminating the cost of film, chemicals and processor equipment. It should not escape one’s mind that digital imaging systems also have their relevant quality control requirements needed for optimal viewing of images.

## CONCLUSION

This study showed that the viewing conditions at Johannesburg Hospital were variably in compliance with guidelines from international organisations. To improve the viewing box uniformity at the Division of Radiology, it was suggested that the viewing boxes be cleaned and the fluorescent lamps be replaced regularly. Furthermore, this study underscores the need of implementing quality control and quality assurance standards in radiographic image viewing, in support of overall optimisation of the radiographic process.

The authors are grateful to Nicholas van der Merwe of Gauteng Provincial Health Department for lending them the Precision Photometer used in this investigation. In addition we would like to acknowledge Professor Debbie van der Merwe, Director of Medical Physics, Johannesburg Hospital for corrections on the manuscript. Ms Queen Letsoalo at the Division of Radiology is acknowledged for her assistance.
